# Medicinal Plants from Mexico, Central America, and the Caribbean Used as Immunostimulants

**DOI:** 10.1155/2016/4017676

**Published:** 2016-03-03

**Authors:** Angel Josabad Alonso-Castro, María del Carmen Juárez-Vázquez, Nimsi Campos-Xolalpa

**Affiliations:** ^1^Departamento de Farmacia, División de Ciencias Naturales y Exactas, Universidad de Guanajuato, 36050 Guanajuato, GTO, Mexico; ^2^Departamento de Productos Naturales, Instituto de Química, Universidad Nacional Autónoma de México, 04510 Ciudad de México, Mexico; ^3^Departamento de Sistemas Biologicos, Universidad Autónoma Metropolitana Unidad Xochimilco, 04960 Ciudad de México, Mexico

## Abstract

A literature review was undertaken by analyzing distinguished books, undergraduate and postgraduate theses, and peer-reviewed scientific articles and by consulting worldwide accepted scientific databases, such as SCOPUS, Web of Science, SCIELO, Medline, and Google Scholar. Medicinal plants used as immunostimulants were classified into two categories: (1) plants with pharmacological studies and (2) plants without pharmacological research. Medicinal plants with pharmacological studies of their immunostimulatory properties were subclassified into four groups as follows: (a) plant extracts evaluated for* in vitro* effects, (b) plant extracts with documented* in vivo* effects, (c) active compounds tested on* in vitro* studies, and (d) active compounds assayed in animal models. Pharmacological studies have been conducted on 29 of the plants, including extracts and compounds, whereas 75 plants lack pharmacological studies regarding their immunostimulatory activity. Medicinal plants were experimentally studied* in vitro* (19 plants) and* in vivo* (8 plants). A total of 12 compounds isolated from medicinal plants used as immunostimulants have been tested using* in vitro* (11 compounds) and* in vivo* (2 compounds) assays. This review clearly indicates the need to perform scientific studies with medicinal flora from Mexico, Central America, and the Caribbean, to obtain new immunostimulatory agents.

## 1. Introduction

The immune system is a complex organization of leukocytes, antibodies, and blood factors that protect the body against pathogens [1]. Innate immunity consists of cells such as lymphocytes, macrophages, and natural killer (NK) cells, which are the first line of host defence [2, 3]. The NK cells lyse pathogens and tumor cells without prior sensitization [4]. Activated macrophages defend the host by phagocytosis, releasing the enzyme lysosomal acid phosphatase, and through the synthesis and release of nitrous oxide (NO) and hydrogen peroxide (H_2_O_2_) [5, 6]. These two components inhibit the mitochondrial respiration and the DNA replication of pathogens and cancer cells [7]. When an infection occurs, macrophages and mast cells immediately release interleukins [2]. The interleukins link the communication between cells of the immune system, facilitating innate immune reactions. Among these cytokines, IL-2 and IL-6 induce the stimulation of cytotoxic T cells and enhance the cytolytic activity of NK cells [8, 9]. Interferon gamma (IFN-*γ*), mainly produced by NK cells, exerts antitumor and antiviral effects, increases antigen presentation and lysosomal activity of macrophages, and promotes the cytotoxic effect of NK cells [10].

Immunodeficiency occurs when there is a loss in the number or function of the immune cells, which might lead to infections and diseases such as cancer [11, 12]. Therefore, the discovery of agents which enhance the immune system represents an attractive alternative to the inhibition of tumor growth and the prevention and treatment of some infections. An immunostimulatory agent is responsible for strengthening the resistance of the body against pathogens. In preclinical and clinical studies, some immunostimulatory medicinal plants (e.g.,* Viscum album* and* Echinacea purpurea*) have increased the immune responsiveness by activating immune cells [3, 11].

In ancient traditional medicine, the term immunostimulant was unknown. In some cases, medicinal plants species that “purify the blood,” “strengthen the body,” and “increase the body's defences” have been used as immunostimulant agents [13, 14].

Some of the* in vitro* and* in vivo* tests used to evaluate the immunostimulatory effects of plant extracts and compounds include the following: (a) proliferation of splenocytes, macrophages, and lymphocytes, (b) phagocytosis, (c) pinocytosis, (d) production of NO and/or H_2_O_2_, (e) NK cell activity, (f) release of IFN-*γ*, IL-2, IL-6, and other interleukins, and (g) lysosomal enzyme activity.* In vivo* studies mainly consist in the induction of an immunosuppressed state in the animals by using (a) chemical agents such as 5-fluorouracil, cyclophosphamide, and methotrexate or (b) biological agents such as tumorigenic cells. All the above-mentioned agents have been extensively studied on inducing immunosuppression [15, 16].

This review provides ethnomedicinal, phytochemical, and pharmacological information about plants and their active compounds used as immunostimulants in Mexico, Central America, and the Caribbean. This information will be useful for developing preclinical and clinical studies with the plants cited in this review.

## 2. Methodology

A literature search was conducted from December 2014 to July 2015 by analyzing the published scientific material on native medicinal flora from Mexico, Central America, and the Caribbean. Academic information from the last five decades that describes the ethnobotanical, pharmacological, and chemical characterization of medicinal plants used as immunostimulants was gathered. The following keywords were used to search for the academic information: plant extract, plant compound, immune system, immunostimulant, immunostimulatory, Mexico, Central America, and the Caribbean. No restrictions regarding the language of publication were imposed, but the most relevant studies were published in Spanish and English. The criteria for the selection of reports in this review were as follows: (i) plants native to Mexico, Central America, and the Caribbean, (ii) plants used in traditional medicine as immunostimulants with or without pharmacological evidence, and (iii) plants and their active compounds with information obtained from a clear source. The immunostimulatory activity of plant extracts or compounds in combination with a known immunostimulant agent (such as lipopolysaccharide, CD3) was omitted in this review.

Medicinal plants used as immunostimulants were classified into two categories: (1) plants with pharmacological studies and (2) plants without pharmacological research. The information on medicinal plants with pharmacological studies was obtained from peer-reviewed articles by consulting the academic databases SCOPUS, Web of Science, SCIELO, Medline, and Google Scholar. Medicinal plants with pharmacological studies of their immunostimulatory properties were subclassified into four groups: (a) plant extracts that have been evaluated for* in vitro* effects, (b) plant extracts with documented* in vivo* effects, (c) active compounds tested using* in vitro* studies, and (d) active compounds that have been assayed in animal models. The information for medicinal plants without pharmacological research was obtained from both undergraduate and postgraduate theses, in addition to peer-reviewed articles, and scientific books.

## 3. Medicinal Plants from Mexico, Central America, and the Caribbean Used as Immunostimulants

We documented 104 plant species belonging to 55 families that have been used as immunostimulants. Of these plants, 28 have pharmacological studies (Table 1), and 76 plants lacked pharmacological research regarding their immunostimulatory activity (Table 6). All plant names and their distributions were confirmed by consulting the Missouri botanical garden (http://www.tropicos.org/). Asteraceae (11 plant species), Fabaceae (8 plant species), and Euphorbiaceae (7 plant species) are the plant families most often used as immunostimulants, including plants with and without pharmacological studies (Tables 1 and 6). We found that 46% of plants used as immunostimulants, with or without pharmacological studies, are also used for the empirical treatment of cancer. This was confirmed, for many plant species, by consulting our previous work [94]. Therefore, we highly recommend evaluating the immunostimulatory effects of medicinal plants used for cancer treatment. Medicinal plants used as immunostimulants are also used for the treatment of diarrhea (23%), cough (18%), and inflammation (18%). Diarrhea and cough are two symptoms associated with gastrointestinal and respiratory infections, respectively. We may therefore infer that immunostimulatory plants may also be used for the treatment and prevention of infections. Medicinal plants used as an antiparasitic agent may treat diseases such as malaria, whereas plants used as antivirals may treat diseases such as measles, smallpox, and others (Tables 1 and 6).

A total of 20 plants, belonging to 15 botanical families, have* in vitro* studies regarding their immunostimulatory effects (Table 2). Furthermore, 8 plant species from 8 botanical families were assessed using* in vivo* assays (Table 3). A total of 11 compounds, isolated from 7 plants, have been tested using* in vitro* assays (Table 4). Only two compounds, isolated from two plants, were studied using* in vivo* models (Table 5).

**Figure 1:**
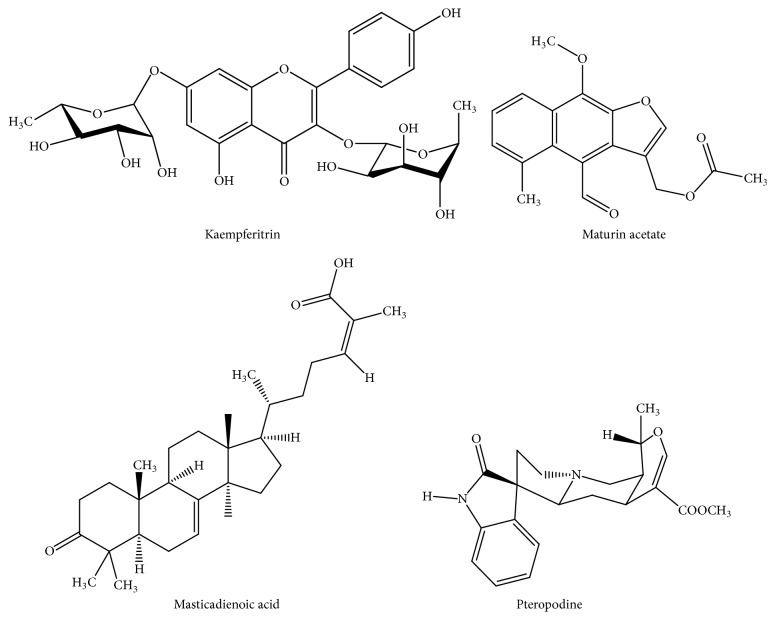
Chemical structures of some compounds with immunostimulatory effects isolated from medicinal plants.

Among the* in vitro* studies,* Lophophora williamsii* was one of the plant species that showed good immunostimulatory effects. This plant tested at 0.18 *μ*g/mL showed a similar activity (2.4-fold, compared to untreated cells) on the proliferation of human primary lymphocytes, compared to the positive control 0.6 *μ*g/mL concanavalin A [26]. Further studies with* Lophophora williamsii*, as well as the isolation and purification of its active compounds, are highly recommended. Among the* in vivo* studies, an ethanol extract from* Phoradendron serotinum* leaves, tested from 1 to 10 mg/kg i.p., showed immunostimulatory effects, in a dose-dependent manner, by increasing the levels of IFN-*γ*, IL-2, and IL-6 in serum from C57BL/6 mice bearing TC-1 tumor [41]. The immunostimulatory effects obtained using* in vitro* studies were confirmed in* in vivo* studies for some plant species such as* Mollugo verticillata*,* Phoradendron serotinum*, and* Petiveria alliacea* and compounds such as maturin acetate (Figure 1). This indicates that these plants and the compound can be metabolized, and their immunostimulatory effects are also shown in animals.

On the other hand, in many works cited in this review, only one concentration or dose was tested. Further studies will be required to obtain the EC_50_ or ED_50_ values, if possible, and analyze whether the plant extracts or compounds induce a concentration/dose-dependent effect. In many studies, a single immunostimulant test is used (e.g., the NO production). Authors are encouraged to perform more than one immunostimulatory test in further studies to provide more information on the immunostimulant effects of plant extracts or compounds. In some cases, the initial screening of the* in vivo* immunostimulatory effects is carried out using immunocompetent mice. Further studies are necessary to be performed on plant extracts and compounds using models of immunosuppressed mice, induced with chemical or biological agents.

## 4. Medicinal Plants Used as Immunostimulants without Pharmacological Studies

We documented 75 medicinal plants used as immunostimulants that lack pharmacological studies (Table 6). Plants from the* Smilax* genus (*S. domingensis*,* S. moranensis*, and* S. spinosa*) and the* Juglans* genus (*J. major*,* J. mollis*, and* J. jamaicensis*) could be an excellent option for the isolation and identification of immunostimulatory agents because compounds isolated from their related species have shown immunostimulatory activity. Smilaxin (1.56 *μ*M), a 30 kDa protein obtained from* Smilax glabra*, increased the proliferation of splenocytes and bone marrow cells with similar activity to the positive control 0.52 *μ*M concanavalin A [95]. A water-soluble polysaccharide, called JRP1, isolated from* Juglans mandshurica* showed* in vivo* immunostimulatory effects by increasing the release of IFN-*γ* and IL-2 in an immunosuppressed model of mice bearing S-180 tumor [96]. Taking this into consideration, further studies with plants from the* Smilax* and* Juglans* genera should be carried out. Furthermore, mistletoe species such as* Phoradendron brachystachyum* and* Psittacanthus calyculathus* could be a good option for discovering immunostimulatory agents since the related species* Phoradendron serotinum* showed good immunostimulatory activity [41]. However, the toxicity of the mistletoe species should be assessed.

## 5. Further Considerations

More ethnobotanical studies are necessary to provide information on medicinal plants used as immunostimulants in Mexico, Central America, and the Caribbean. The ethnomedicinal information of plant species will be updated with these studies.

The toxicity of plant species cited in this review should also be assessed. For instance,* Xanthium strumarium* is considered a toxic plant. Recently, it was described that this plant induces hepatotoxicity [97]. On the contrary,* Hymenaea courbaril* was shown to lack genotoxic and mutagenic effects [98]. Toxicological studies are necessary to provide safety in the use of plant extracts and their compounds in clinical trials.

To our knowledge, there are no pharmacokinetic studies carried out with plant compounds cited in this review. This might be due to (a) the lack of established methodologies for their quantitation, (b) the quantity of the obtained compound being not enough to carry out a pharmacokinetic study, and (c) many plants extracts not being chemically characterized, and there is no main metabolite for its quantification using HPLC. Further pharmacokinetic studies will provide additional pharmacological information prior to carrying out clinical trials. The isolation and elucidation of the structure of bioactive principles should also be encouraged.

Eight percent of medicinal plants listed in this review are classified as endangered. In the order of most endangered,* Juglans jamaicensis*,* Cedrela odorata*, and* Lophophora williamsii* are cataloged as vulnerable, whereas* Taxodium mucronatum*,* Rhizophora mangle*,* Eysenhardtia polystachya*,* Cordia alliodora*, and* Hymenaea courbaril* are cataloged as of least concern [99]. For instance,* Lophophora williamsii* (peyote) is a species that has been overexploited because of its high content of hallucinogenic alkaloids. The conservation of these species, as well as their habitats, should be encouraged by national and international programs to preserve biodiversity.

**Figure 2:**
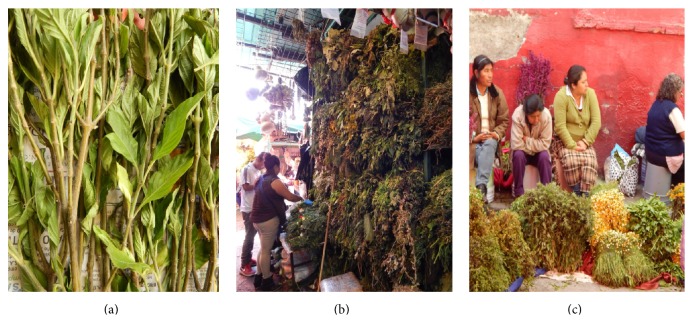
Trade of medicinal plants used as immunostimulants in Mexico City. (a)* Justicia spicigera* was the most cited plant species used as immunostimulatory agent. (b and c) Traditional markets in Mexico City, showing the sellers of medicinal plants called hierberos or yerbateros.

There is null or limited information regarding the trade of medicinal plants used as immunostimulants. Therefore, we performed direct interviews (*n* = 45) with local sellers of medicinal plants in Mexico, called “hierberos” or “yerbateros” in 7 different markets (Portales, Sonora, Xochimilco, Milpa Alta, Tlahuac, and Ozumba) located in Mexico City and the metropolitan area (Figure 2). Two of the markets are located in Xochimilco. In order of importance, the most recommended plant species used as immunostimulants are* Justicia spicigera*,* Polygonum aviculare*,* Carlowrightia cordifolia, Amphipterygium adstringens, Uncaria tomentosa,* and others. It was interesting to find that 85% of yerbateros recommended the use of* Justicia spicigera* as immunostimulant (Figure 2(a)). Its way of preparation consists of the following: four or five branches and leaves are boiled with 1 L of water during 30 min. The recommended administration is 3 times daily. The rest of plant species were cited by less than 10% of yerbateros.

The demand for medicinal plants used as immunostimulants clearly indicates that these plant species are a current topic of interest. This indicates that ethnobotanical knowledge is a valuable tool, which supports the selection of plants to carry out pharmacological studies. Some of the medicinal plants cited in our survey have been pharmacologically investigated.* Carlowrightia cordifolia* showed poor immunostimulatory effects [17].* Amphipterygium adstringens* showed* in vivo* immunostimulatory effects [51], whereas masticadienonic acid (Figure 1), its active compound at 0.001 *μ*M, increased the NO production (1.8 fold) with higher activity compared to 0.001 *μ*M ursolic acid (1.4 fold) [55].* Uncaria tomentosa* showed* in vitro* immunostimulatory effects [50], whereas pteridine (Figure 1), its active compound, tested at 600 mg/kg i.p., increased the lymphocyte proliferation in immunocompetent mice [59].* Justicia spicigera* and kaempferitrin (Figure 1), its active compound, showed* in vitro* immunostimulatory effects [14, 54]. Nevertheless, the* in vivo* immunostimulatory effects remain to be performed with* Justicia spicigera*, kaempferitrin, and masticadienonic acid. The molecular mechanism by which this plant and the compounds exert their immunostimulatory effects should also be assessed.

Finally, this review highlights the need to perform pharmacological, phytochemical, toxicological, and ethnobotanical studies with medicinal flora, from Mexico, Central America, and the Caribbean, to obtain new immunostimulatory agents.

## Figures and Tables

**Table 1:** Medicinal plants with pharmacological evidence of their immunostimulant effects.

Family	Scientific name	Common name	Plant part	Other popular uses	Reference
Acanthaceae	*Carlowrightia cordifolia *A. Gray	Arnica	Lv	AI	[17]
	*Justicia spicigera *Schltdl.	Muicle	Lv	DB, CA	[18]
Anacardiaceae	*Amphipterygium adstringens* (Schltdl.) Standl.	Cuachalalate	Bk	SA, DG, CA	[19]
Asteraceae	*Bidens pilosa *L.	Aceitilla	Wp	DB, DI, SA, CA	[20]
	*Psacalium peltatum *(Kunth) Cass.	Matarique	Rt	WH, BP, CA	[21]
	*Tridax procumbens *L.	Ghamra	Ap	WH	[22]
	*Xanthium strumarium *L.	Guizazo de caballo	Rt	DU, CA	[23]
Bignoniaceae	*Tabebuia chrysantha* (Jacq.) G. Nicholson	Guayacan	Bk	AI, DB, SA	[24]
Cactaceae	*Lophocereus schottii *(Engelm.) Britton & Rose	Garambullo	Sm	CO, DB, SA, CA	[25]
	*Lophophora williamsii* (Lem. ex Salm-Dyck) J. M. Coult.	Peyote	Tb	BP, CA	[26]
Caricaceae	*Carica papaya* L.	Papaya	Fr	SA, DG, DI, CA	[27]
Euphorbiaceae	*Euphorbia cotinifolia *L.	Palito lechero	Latex	AI	[28]
	*Euphorbia hirta *L.	Tártago de jardín	Ap	AV	[29]
	*Euphorbia pulcherrima *Willd. ex Klotzsch	Nochebuena	Ap	AI, CO, FL, CA	[28]
	*Hura crepitans *L.	Ceiba	Lv	AI	[28]
Fabaceae	*Hymenaea courbaril *L.	Guapinol	Bk	DU, AP	[30]
	*Mucuna urens *(L.) Medik.	Tortera	Bk	DU	[31]
	*Phaseolus vulgaris *L.	Frijol	Sd	DI, BP	[32]
Hypericaceae	*Hypericum perforatum* L.	Hierba de San Juan	Wp	DP, WH	[33]
Lauraceae	*Persea americana* Mill.	Aguacate	Lv	AH, BP, WH, CA	[34]
Molluginaceae	*Mollugo verticillata *L.	Hierba de la arena	Ap	AI	[35]
Nyctaginaceae	*Bougainvillea × buttiana* Holttum & Standl.	Bugambilia	Fw	SA, CO	[36]
Phyllanthaceae	*Phyllanthus niruri *L.	Chancapiedra	Ap	AI, DU, CA	[37]
Phytolaccaceae	*Petiveria alliacea *L.	Anamú	Ap	AI, SA, BP, CA	[38]
Plantaginaceae	*Plantago virginica* L.	Platano	Lv	AI	[39]
Rubiaceae	*Uncaria tomentosa *(Willd.) DC.	Uña de gato	Bk	AV, CA	[40]
Santalaceae	*Phoradendron serotinum *(Raf.) M. C. Johnst.	Muerdago	Lv	DB, CA	[41]
Talinaceae	*Talinum triangulare *(Jacq.) Willd.	Espinaca	Lv	CA, AV, DB	[42]
Urticaceae	*Phenax rugosus* (Poir.) Wedd.	Parietaria	Wp	WH, AV	[43]

Other popular uses: AP: antiparasitic; AI: anti-inflammatory; AV: antiviral; BP: body pain; CA: cancer; CO: cough; DG: digestive; DI: diarrhea; DU: diuretic; DP: depression; FL: flu; SA: stomachache; TB: tuberculosis; WH: wound healing. Plant part: Ap: aerial parts; Bk: bark; Br: branches; Fr: fruit; Lv: leaves; Fw: flower; Rb: root bark; Rt: root; Sd: seeds; Sm: stem; Tb: tubercle; Wp: whole plant.

**Table 2:** Plant extracts with immunostimulatory effects tested using *in vitro* assays.

Family	Scientific name	Plant part	Extract	Range of concentration tested *μ*g/mL	Immunostimulatory effects, compared to untreated control [duration of the experiment]	Reference
Acanthaceae	*Carlowrightia cordifolia *A. Gray	Lv	Hex	13.3 (mg/mL)	NO production (2.5-fold) at 13.3 mg/mL [48 h] in human primary peritoneal macrophage	[17]
	*Justicia spicigera *Schltdl.	Lv	EtOH	10–200	Induction of phagocytosis (0.4-fold) at 200 *μ*g/mL [48 h] by human primary lymphocytes against *Saccharomyces cerevisiae* NO production (6.4-fold) in murine primary macrophages and H_2_O_2_ release (8.5-fold) at 200 *μ*g/mL with murine monocyte–macrophages cocultured with *Saccharomyces cerevisiae* [48 h] Proliferation of human primary lymphocytes (0.4-fold) at 200 *µ*g/mL [48 h]	[14]
Asteraceae	*Bidens pilosa *L.	Wp	H_2_O	500	Increased on IFN-*γ* promoter (1.9-fold) in Jurkat T cells at 500 *µ*g/mL [72 h]	[44]
	*Xanthium strumarium *L.	Wp	H_2_O	10–100	Proliferation of murine primary lymphocytes (13-fold) at 100 *µ*g/mL [44 h]	[45]
Cactaceae	*Lophophora williamsii* (Lem. ex Salm-Dyck) J. M. Coult.	Tb	MeOH	0.18–18	Proliferation of murine primary lymphocytes (2.5-fold) at 0.18–1.8 *μ*g/mL [72 h] NO production (3-fold) at 18 *μ*g/mL using murine peritoneal macrophages [72 h]	[26]
Caricaceae	*Carica papaya *L.	Lv	H_2_O	1.25–5 (mg/mL)	Production of IFN-*γ* (2.0-fold), IL-12 p40 (2.0-fold) in human primary lymphocytes at 1.25 mg/mL [24 h]	[46]
Euphorbiaceae	*Euphorbia cotinifolia *L.	Latex	—	25	Proliferation of human primary lymphocytes (1.6-fold) at 25 *μ*g/mL [66 h]	[47]
	*Euphorbia hirta *L.	Ap	EtOH	0.06–500 (mg/mL)	Induction of phagocytosis of *Candida albicans *(2.0-fold) by primary murine macrophages at 500 mg/mL [1 h]	[29]
	*Euphorbia pulcherrima *Willd. ex Klotzsch	Lv	Hex : DCM : MeOH (2 : 1 : 1)	25	Proliferation of human primary lymphocytes (6.5-fold) at 25 *μ*g/mL [66 h]	[47]
	*Hura crepitans *L.	Lv	Hex : DCM : MeOH (2 : 1 : 1)	25	Proliferation of human primary lymphocytes (0.85-fold) at 25 *μ*g/mL [66 h]	[47]
Hypericaceae	*Hypericum perforatum* L.	Wp	H_2_O	750	Proliferation of murine primary lymphocytes (1.6-fold) at 750 *μ*g/mL [18 h]	[48]
Lauraceae	*Persea americana* Mill.	Lv	MeOH	3.91–250	Proliferation of murine primary lymphocytes (1.6-fold) at 250 *μ*g/mL [48 h]	[39]
Molluginaceae	*Mollugo verticillata *L.	Ap	EtOH	25	NO production (1.6-fold) at 25 *μ*g/mL using murine peritoneal primary macrophages cocultures with *Mycobacterium tuberculosis* [48 h]	[44]
Nyctaginaceae	*Bougainvillea × buttiana* Holttum & Standl.	Fw	EtOH	2.9–290	H_2_O_2_ production (0.4-fold) at 2.9 *μ*g/mL with murine primary peritoneal macrophages [24 h] Proliferation of murine primary peritoneal macrophages (0.6-fold) at 29 *μ*g/mL [48 h] NO production (2.4-fold) at 290 *μ*g/mL with murine primary peritoneal macrophages [48 h]	[36]
Phyllanthaceae	*Phyllanthus niruri *L.	Lv	Hex : DCM : MeOH (2 : 1 : 1)	25	Proliferation of human primary lymphocytes (1.3-fold) at 25 *μ*g/mL [66 h]	[47]
Phytolaccaceae	*Petiveria alliacea *L.	Ap	H_2_O	25	Production of IL-6 (100-fold), IL-10 (14-fold), and IL-8 (12-fold) in dendritic cells at 25 *μ*g/mL [48 h]	[49]
Plantaginaceae	*Plantago virginica* L.	Lv	MeOH	3.91–250	Proliferation of murine primary lymphocytes at 250 *μ*g/mL (1.6-fold) [48 h]	[39]
Rubiaceae	*Uncaria tomentosa *(Willd.) DC.	Rb	H_2_O	0.32–320	NO production (1.5-fold) at 320 *μ*g/mL using murine primary peritoneal macrophages [48 h] Production of IL-6 (7.2-fold) at 320 *μ*g/mL in murine primary peritoneal macrophages [24 h]	[50]
Santalaceae	*Phoradendron serotinum *(Raf.) M. C. Johnst.	Lv	EtOH	1–50	Proliferation of RAW 264.7 macrophages (0.2-fold) and murine primary splenocytes (0.3-fold) at 50 *μ*g/mL [48 h] Lysosomal enzyme activity (0.2-fold) at 50 *μ*g/mL using RAW 264.7 macrophages [48 h] Stimulation of NK cell activity (7.1-fold) at 50 *μ*g/mL using murine primary splenocytes cocultured with K562 cells [48 h] Production of IFN-*γ* (1.6-fold), IL-2 (1.4-fold), and IL-6 (1.3-fold) at 50 *μ*g/mL using murine primary splenocytes cocultured with K562 cells [48 h]	[41]
Talinaceae	*Talinum triangulare *(Jacq.) Willd.	Sm	EtOH	100–1000	Proliferation of human primary lymphocytes (2-fold) at 1000 *μ*g/mL [72 h] NO production (4-fold) at 1000 *μ*g/mL [72 h] Production of IFN-*γ* (16-fold) at 500 *μ*g/mL in human primary lymphocytes [72 h]	[42]

Solvent used for the extract:  Hex: hexane; DCM: dichloromethane; MeOH: methanol; EtOH: ethanol; H_2_O: aqueous. Plant part:  Rb: root bark; Tb: tubercle; Lv: leaves; Wp: whole plant.

**Table 3:** Plant extracts with immunostimulatory effects tested using *in vivo* assays.

Family	Scientific name	Plant part	Extract	Model of immunosuppression and duration of the experiment [range of dose tested]	Immunostimulatory effects (compared to immunosuppressed mice)	Reference
Anacardiaceae	*Amphipterygium adstringens* (Schltdl.) Standl.	Bk	H_2_O	BALB/c mice bearing lymphoma L5178Y for 10 days [10 mg/kg p.o.]	Proliferation of splenocytes (2.0-fold) at 10 mg/kg	[51]
Asteraceae	*Tridax procumbens *L.	Ap	H_2_O	Immunocompetent Swiss mice for 6 days [250 and 500 mg/kg i.p.]	Increase of leukocyte number (1.4-fold) at 500 mg/kg Increase in phagocytic index (0.3-fold) at 500 mg/kg	[22]
Bignoniaceae	*Tabebuia chrysantha* (Jacq.) G. Nicholson	Lv	H_2_O : EtOH (1 : 1)	Wistar rats immunized with sheep red blood cells for 17 days [1000 mg/kg p.o.]	Increase of leucocyte number (1.2-fold) at 1000 mg/kg	[24]
Cactaceae	*Lophocereus schottii *(Engelm.) Britton & Rose	Sm	EtOH	BALB/c mice bearing lymphoma L5178Y for 22 days [10 mg/kg p.o.]	Proliferation of lymphocytes (0.2-fold) at 10 mg/kg	[25]
Molluginaceae	*Mollugo verticillata *L.	Ap	EtOH	Mice inoculated with 0.1 mg Bacillus Calmette–Guérin for 7 days [500 mg/kg p.o.]	NO production (3.1-fold) at 500 mg/kg	[52]
Phytolaccaceae	*Petiveria alliacea *L.	Ap	H_2_O	BALB/c mice treated with 5-fluorouracil for 4 days [400 and 1200 mg/kg p.o.]	Increase of leukocyte number (1.4-fold) at 1200 mg/kg	[53]
Santalaceae	*Phoradendron serotinum *(Raf.) M. C. Johnst.	Lv	EtOH	C57BL/6 mice bearing TC-1 tumor for 25 days [1–10 mg/kg i.p.]	Production of IFN-*γ* (1.3-fold), IL-2 (2.1-fold), and IL-6 (2.1-fold) at 10 mg/kg	[41]
Urticaceae	*Phenax rugosus* (Poir.) Wedd.	Lv	H_2_O : EtOH (1 : 1)	Wistar rats immunized with sheep red blood cells for 17 days [1000 mg/kg p.o.]	Increase of leucocyte number (1.5-fold) at 1000 mg/kg	[24]

Solvent used for the extract: EtOH: ethanol; H_2_O: aqueous. Plant part: Rb: root bark; Tb: tubercle; Lv: leaves; Wp: whole plant; Ap: aerial parts; Sm: stem; Bk: bark.

**Table 4:** *In vitro* immunostimulatory effects of plant compounds.

Family	Scientific name	Compound	Group	Range of concentration tested *µ*M	Immunostimulatory effects, compared to untreated control [duration of the experiment]	Reference
Acanthaceae	*Justicia spicigera* Schtdl.	Kaempferitrin	Flavonoid	1–25	Induction of phagocytosis (0.4-fold) at 200 *µ*g/mL using RAW 264.7 macrophages [48 h] Induction of lysosomal enzyme activity (0.5-fold) at 25 *µ*M with RAW 264.7 macrophages [48 h] Increase of NK cell activity (10-fold) at 25 *µ*M with RAW 264.7 macrophages cocultured with K562 cells [48 h]	[54]
Anacardiaceae	*Amphipterygium adstringens* (Schltdl.) Standl.	Masticadienonic acid	Triterpenoid	0.001–10	NO production (1.8-fold) [72 h] at 0.001 *µ*M in murine primary peritoneal macrophages	[55]
		3*α*-Hydroxymasticadienolic acid	Triterpenoid	0.001–10	NO production (1.7-fold) [72 h] at 1 *µ*M in murine primary peritoneal macrophages	
		24,25S-dihydromasticadienonic acid	Triterpenoid	0.001–10	NO production (1.3-fold) [72 h] at 0.01 *µ*M in murine primary peritoneal macrophages	
		Masticadienolic acid	Triterpenoid	0.001–10	NO production (1.6-fold) [72 h] at 0.1 *µ*M in murine primary peritoneal macrophages	
Asteraceae	*Bidens pilosa* L.	Centaurein Centaureidin	Flavonoid Flavonoid	EC_50_ = 0.14 *µ*M EC_50_ = 2.5 *µ*M	Increase on IFN-*γ* promoter in Jurkat T cells [72 h]	[44]
	*Psacalium peltatum *(Kunth) Cass.	Maturin acetate	Sesquiterpene	1–25	Increase of NK cell activity (7-fold) at 25 *µ*M using murine primary splenocytes cocultured with K562 cells [48 h] Induction of lysosomal enzyme activity (0.2-fold) at 25 *µ*M using RAW 264.7 macrophages [48 h] Proliferation of RAW 264.7 macrophages and murine primary splenocytes (0.2-fold, each) at 25 *µ*M [48 h]	[56]
Fabaceae	*Hymenaea courbaril* L.	Xyloglucan	Polysaccharide	0.1–50	NO production (2.1-fold) at 0.25 *µ*M with murine primary peritoneal macrophages [48 h]	[57]
	*Mucuna urens *(L.) Medik.	Xyloglucan	Polysaccharide	0.06–3.2	NO production (1.4-fold) at 0.16 *µ*M with murine primary peritoneal macrophages [48 h]	
	*Phaseolus vulgaris *	Pectic polysaccharide	Polysaccharide	0.07–1.12	Murine primary splenocytes proliferation (2.5-fold) at 1.12 *µ*M [72 h] Murine primary thymocyte proliferation (2.1-fold) at 0.14 *µ*M [72 h]	[58]

**Table 5:** *In vivo* immunostimulatory effects of plant compounds.

Family	Scientific name	Compound	Group	Model of immunosuppression and duration of the experiment [range of dose tested]	Immunostimulatory effects (compared to immunosuppressed mice)	Reference
Asteraceae	*Psacalium peltatum *(Kunth) Cass.	Maturin acetate	Sesquiterpene	BALB/c mice treated with 100 mg/kg cyclophosphamide for 14 days [10–50 mg/kg i.p.]	Production of IFN-*γ* (1.4-fold) and IL-2 (1.8-fold)	[56]
Rubiaceae	*Uncaria tomentosa *(Willd.) DC.	Pteropodine	Alkaloid	Immunocompetent mice for 4 days [100–600 mg/kg i.p.]	Lymphocyte proliferation (1.6-fold) at 600 mg/kg	[59]

**Table 6:** Medicinal plants used as immunostimulants with no pharmacological studies.

Family	Scientific name	Common name	Plant part	Other popular uses	Reference
Adoxaceae	*Sambucus mexicana *C. Presl ex DC.	Sauco	Lv	AI, CO, DU	[60]
Agavaceae	*Agave americana *L.	Maguey	Ap	DU, CA	[61]
	*Agave salmiana *Otto ex Salm-Dyck	Agave	Ap	DU, CA	[19]
	*Agave tequilana* F. A. C. Weber	Agave	Ap	DG	[62]
	*Furcraea tuberosa* (Mill.) W. T. Aiton	Maguey	Rt	AI	[31]
Amaranthaceae	*Chenopodium ambrosioides *L.	Epazote	Lv	AP, DI, CA	[63]
	*Chenopodium berlandieri *Moq.	Epazote	Lv	BR, AP	[63]
	*Chenopodium incisum* Poir.	Epazote zorrillo	Lv	AP, DU	[63]
	*Iresine ajuscana* Suess. & Beyerle	Iresine	Lv	AI	[13]
Anacardiaceae	*Spondias mombin *L.	Jobo	Fr	WH, DI	[64]
Asteraceae	*Austroeupatorium inulifolium* (Kunth) R. M. King & H. Rob.	Salvia amarga	Wp	CO	[65]
	*Bidens aurea *(Aiton) Sherff	Aceitilla	Wp	DB, DI, SA	[66]
	*Mikania cordifolia *(L. f.) Willd.	Trepadora	Lv	AI, CO, BP	[67]
	*Neurolaena lobata* (L.) Cass.	Burrito	Rt	BP, DB, CA, AP	[67]
	*Pterocaulon alopecuroides* (Lam.) DC.	Varita pienegro	Wp	AV, CA	[68]
	*Sanvitalia ocymoides *DC.	Ojo de gallo	Wp	DI, SA	[69]
	*Tagetes lucida* Cav.	Pericón	Ap	SA, DP, CA	[33]
Bignoniaceae	*Crescentia alata *Kunth	Huaje	Fr	TB, CA, DI	[70]
	*Parmentiera aculeata *(Kunth) Seem.	Cuajilote	Ap	DB, BP, DU, CO, DI	[60]
	*Tecoma stans *(L.) Juss. ex Kunth	Tronadora	Ap	DB, DU, CA	[71]
Bixaceae	*Bixa orellana *L.	Achiote	Sd	CA, WH, DU	[68]
Bromeliaceae	*Ananas comosus *(L.) Merr.	Pineapple	Fr	DB, AH, CA	[72]
Burseraceae	*Bursera copallifera *(DC.) Bullock	Copal	Ap	AI, CA	[73]
	*Bursera fagaroides* (Kunth) Engl.	Palo xixote	Bk	SA, CA	[74]
	*Bursera simaruba* (L.) Sarg.	Palo mulato	Lv	CO, SA, CA	[67]
Commelinaceae	*Zebrina pendula *Schnizl.	Hierba de pollo	Lv	BP, WH, DB, CA	[43]
Cordiaceae	*Cordia alliodora* (Ruiz & Pav.) Oken	Aguardientillo	Lv	TB, WH	[67]
	*Varronia globosa *Jacq.	Yerba de la sangre	Ap	DU	[23]
Costaceae	*Costus arabicus *L.	Caña Guinea	Ap	AI	[75]
Cupressaceae	*Taxodium mucronatum *Ten.	Ahuehuete	Br	DI	[76]
Gesneriaceae	*Moussonia deppeana* (Schltdl. & Cham.) Hanst.	Tlalchichinole	Ap	WH, DI	[19]
Euphorbiaceae	*Acalypha phleoides *Cav.	Hierba del cáncer	Ap	CA, DI	[76]
	*Cnidoscolus aconitifolius *(Mill.) I. M. Johnst.	Chaya	Lv	DB, CA	[28]
	*Codiaeum variegatum *(L.) Rumph. ex A. Juss.	Croton	Lv	DI	[28]
Equisetaceae	*Equisetum laevigatum* A. Braun	Cola de caballo	Ap	DU	[77]
Fabaceae	*Desmodium molliculum *(Kunth) DC.	Manayupa	Ap	DU, WH	[40]
	*Eysenhardtia polystachya *(Ortega) Sarg.	Palo dulce	Lv	DU, DB, WH, CA	[78]
	*Haematoxylum brasiletto* H. Karst.	Palo de Brasil	Bk	CO, DI	[19]
	*Senna reticulata *(Willd.) H. S. Irwin & Barneby	Barajo	Ap	DB, WH	[43]
	*Zornia thymifolia *Kunth	Hierba de la vibora	Wp	DI, BP	[66]
Juglandaceae	*Juglans jamaicensis *C. DC.	Palo de nuez	Bk	WH, AP	[31]
	*Juglans major *(Torr.) A. Heller	Nogal	Lv	DU, AP, WH, CA	[76]
	*Juglans mollis *Engelm.	Nuez de caballo	Ap	WH, BP	[79]
Krameriaceae	*Krameria grayi *Rose & J. H. Painter	Zarzaparrilla	Wp	DU	[20]
Lamiaceae	*Salvia regla *Cav.	Salvia	Lv	WH	[63]
	*Satureja macrostema *(Moc. & Sessé ex Benth.) Briq.	Té de monte	Lv	CO	[80]
Lauraceae	*Cinnamomum pachypodum* (Nees) Kosterm.	Laurel	Ap	AP	[63]
Loranthaceae	*Psittacanthus calyculatus *(DC.) G. Don	Muerdago	Ap	CA, WH	[76]
Meliaceae	*Cedrela odorata *L.	Cedro	Bk	TB, DI	[37]
Myrtaceae	*Psidium guajava *L.	Guayaba	Ap	AI, DI, CA	[81]
Moraceae	*Brosimum alicastrum *Sw.	Ojite	Lv	TB, FL	[82]
Musaceae	*Musa sapientum* L.	Banana	Fr	DI, DG	[83]
Onagraceae	*Ludwigia peploides *(Kunth) P. H. Raven	Clavo de la laguna	Ap	CO	[43]
Orobanchaceae	*Castilleja tenuiflora *Benth.	Cola de borrego	Ap	WH, CO, DI, CA	[84]
Papaveraceae	*Bocconia frutescens *L.	Gordolobo	Lv	CO, SA, CA	[35]
Passifloraceae	*Turnera diffusa *Willd.	Damiana	Lv	CO, DI, CA	[79]
Piperaceae	*Piper auritum *Kunth	Acoyo	Lv	SA, CO, DI	[85]
Polemoniaceae	*Loeselia mexicana *(Lam.) Brand	Espinosilla	Ap	DI, DU	[86]
Polygonaceae	*Polygonum aviculare* L.	Sanguinaria	Ap	DI, BR, DU, CA	[76]
Polypodiaceae	*Polypodium polypodioides* (L.) Watt	Helecho de resurrección	Lv	AP	[31]
	*Serpocaulon triseriale* (Sw.) A. R. Sm.	Calaguala	Rt	WH, AH	[87]
Rhizophoraceae	*Rhizophora mangle *L.	Mangle rojo	Bk	DI, DB, CA	[23]
Rubiaceae	*Hamelia patens *Jacq.	Escobetilla	Lv	AI, BP, CA	[67]
Salicaceae	*Salix humboldtiana *Willd.	Sauce criollo	Rt	AI, TB	[88]
	*Zuelania guidonia *(Sw.) Britton & Millsp.	Guaguasí	Bk	WH, CA	[23]
Selaginellaceae	*Selaginella lepidophylla* (Hook. & Grev.) Spring	Doradilla	Wp	DU, CO, CA	[86]
Smilacaceae	*Smilax domingensis* Willd.	Zarzaparrilla	Rt	DI, SA	[89]
	*Smilax moranensis *M. Martens & Galeotti	Zarzaparrilla	Wp	DU, CO	[66]
	*Smilax spinosa *Mill.	Zarzaparrilla	Wp	BP, CA	[90]
Solanaceae	*Lycopersicon esculentum* Mill.	Jitomate	Fr	CO, CA	[13]
	*Solanum americanum *Mill.	Hierba mora	Lv	BP, WH, CA	[91]
Urticaceae	*Urera baccifera *(L.) Gaudich. ex Wedd.	Chichicate	Rt	DU, AI, BP	[23]
Verbenaceae	*Verbena litoralis* Kunth	Verbena negra	Lv	SA, CO, AH	[92]
Viscaceae	*Phoradendron brachystachyum *(DC.) Nutt.	Muerdago	Ap	DB, CA	[93]
Vitaceae	*Cissus sicyoides *L.	Tripa de Judas	Lv	BP, WH, AI, CA	[85]

AP: antiparasitic; AI: anti-inflammatory; AV: antiviral; BP: body pain; CA: cancer; CO: cough; DG: digestive; DI: diarrhea; DU: diuretic; DP: depression; FL: flu; SA: stomachache; TB: tuberculosis; WH: wound healing. Plant part: Ap: aerial parts; Bk: bark; Br: branches; Fr: fruit; Lv: leaves; Fw: flower; Rb: root bark; Rt: root; Sd: seeds; Sm: stem; Tb: tubercle; Wp: whole plant.
